# Challenges in implementing individualized medicine illustrated by antimetabolite therapy of childhood acute lymphoblastic leukemia

**DOI:** 10.1186/1559-0275-8-8

**Published:** 2011-06-03

**Authors:** Jacob Nersting, Louise Borst, Kjeld Schmiegelow

**Affiliations:** 1 Pediatric Oncology Research Laboratory, JMC-5704, Copenhagen University Hospital Rigshospitalet, Blegdamsvej 9, DK-2100 Copenhagen; 2 The Faculty of Medicine, Institute of Gynecology, Obstetrics and Pediatrics, University of Copenhagen, Denmark

**Keywords:** individualized medicine, acute lymphoblastic leukemia, maintenance therapy, clinical implementation

## Abstract

Predicting the response to medical therapy and subsequently individualizing the treatment to increase efficacy or reduce toxicity has been a longstanding clinical goal. Not least within oncology, where many patients fail to be cured, and others are treated to or beyond the limit of acceptable toxicity, an individualized therapeutic approach is indicated. The mapping of the human genome and technological developments in DNA sequencing, gene expression profiling, and proteomics have raised the expectations for implementing genotype-phenotype data into the clinical decision process, but also multiplied the complex interaction of genetic and other laboratory parameters that can be used for therapy adjustments. Thus, with the advances in the laboratory techniques, post laboratory issues have become major obstacles for treatment individualization. Many of these challenges have been illustrated by studies involving childhood acute lymphoblastic leukemia (ALL), where each patient may receive up to 13 different anticancer agents over a period of 2-3 years. The challenges include i) addressing important, but low-frequency outcomes, ii) difficulties in interpreting the impact of single drug or single gene response data that often vary across treatment protocols, iii) combining disease and host genomics with outcome variations, and iv) physicians' reluctance in implementing potentially useful genotype and phenotype data into clinical practice, since unjustified downward or upward dose adjustments could increase the of risk of relapse or life-threatening complications. In this review we use childhood ALL therapy as a model and discuss these issues, and how they may be addressed.

## Introduction

### Individualized medicine

In individualized medicine, physicians seek to balance treatment to obtain optimal clinical effect and minimal adverse reactions by taking patient variability into consideration. Drug dosing has traditionally been adjusted by age, weight or side effects. Thus, in its broadest sense, individualized medicine is not new, but the options and perspectives have become vastly expanded and scientifically established within the last decade [1]. The increased focus largely reflects the expanded number of potential adjustment parameters, including single nucleotide polymorphisms (SNPs) available with the completion of the human genome project and the potential of such markers in predicting patient responses. Interest has focused on variants in (or haplotypes linked to) genes involved in drug absorption, metabolism, transport, and excretion or in drug target pathways. However, variants not related to pharmacogenetics may also be important. In ALL for example, variants of genes encoding proteases, angiogenic factors, hematopoietic cytokines, bone marrow stroma factors, or structural proteins in epithelia may influence disease progression, expansion, or susceptibility to specific toxicities. Technical advances in proteomics and pharmaceutical measurements or *in-vitro *sensitivity testing provide another set of potential adjustment parameters.

The clinical perspectives of individualized medicine have been emphasized and outlined in numerous publications, but in spite of extensive research within almost all areas of medicine, few outcome predictors are implemented in routine clinical decision-making [2]. Hence, re-evaluation of the strategies and feasibility of individualized medicine is warranted to identify clinical settings and logistic requirements, where the expectations are likely to be met.

### Treatment, disease, and host interactions

**Figure 1:**
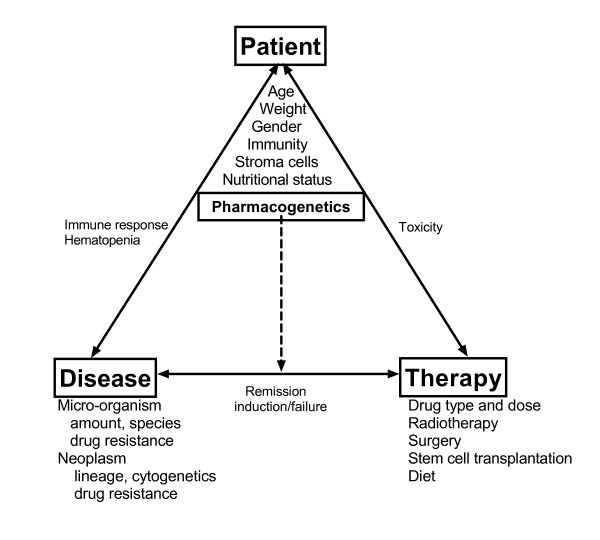
**The applied therapy affects disease and patient leading to treatment failure or cure, and side effects, respectively**. This in term may lead to therapy changes. For drugs with high therapeutic indices, therapy modifications is mainly determined by the response of the disease, whereas for drugs with low therapeutic indices, feedback through both the therapy-patient axis and the therapy-patient axis is likely to modify the therapy. Patient and disease interact directly via immune responses, bone marrow stroma support of leukemia, and suppression of hematopoesis etc. In addition to affecting the therapy-patient interaction, the patient's pharmacogenetic profile affects the therapy-disease axis through its effect on drug clearance, metabolism, distribution etc.

The therapeutic outcome of any disease is determined by the interaction between the patient, the disease, and the therapy (figure 1). The relative impact of patient and disease variants differs depending on the clinical setting.

Many antibiotics (e.g. penicillins) are characterized by high therapeutic indices. Thus, relatively high doses may be administered with a low risk of side effects, and patient variability in drug metabolism can be overcome by accepting very high exposure to some patients in order to ensure sufficient exposure to all. In such cases, the treatment outcome is primarily determined by the therapy-disease interaction, i.e. the drug resistance of the invading microorganism. Accordingly, benefits of individualized medicine are expected to be modest and mostly financial, e.g. if high doses of expensive drugs can be avoided.

The opposite is the case in oncology, where most patients are treated to the limit of acceptable toxicity due to the low therapeutic indices of most anticancer agents and a significant fraction of the patients are treated beyond this limit and experience serious late effects or even deaths due to toxicities [3,4]. Hence, in addition to the therapy-disease axis, variations in the therapy-patient interaction may have substantial effect on treatment response. Previously, the focus was primarily on the effect of a specific treatment on the disease, and treatment failures were generally regarded to represent *resistant disease*. However, many studies have indicated that in childhood ALL, host variations in drug disposition determined by inherited genetic variants may, as frequently as truly resistant disease, lead to treatment failures [3]. In this report we will use antimetabolite-based therapy of childhood ALL to illustrate the challenges we face in individualized medicine, and how they can be addressed.

### Childhood acute lymphoblastic leukemia

In the industrialized countries cancer is the most common medical cause of death in children above the age of 1.0 year and ALL is the most common cancer in childhood. Over the last decades the outcome for children with ALL has changed dramatically from being an almost universally fatal disease to approximately 80% cure rates by first-line therapy owing mainly to intensified treatment made possible through better supportive care and willingness to accept more toxicity [5,6]. Childhood ALL therapy consists of 5 treatment phases: induction, consolidation and re-intensification, CNS-directed treatment, and antimetabolite-based maintenance therapy with 6-mercaptopurine (6MP) and methotrexate (MTX), which is continued until 2-3 years from diagnosis and believed to be of major importance for the improved cure rates [7].

The cytotoxicity of MTX relies on cellular depletion of tetrahydrofolates leading to inhibition of nucleotide *de novo *synthesis and amino acid metabolism [8,9]. Upon intake, 6MP may become inactivated through methylation by thiopurine methyltransferase (TPMT). Some methylated 6MP metabolites (e.g. 6-Methylthioinosine-monophsophate) also inhibit nucleotide *de novo *synthesis, however, the main cytotoxic effect relies on the purine salvage pathway and kinase-mediated multi-step conversion of 6MP into 6-thioguanosine nucleotides (6TGN), which are subsequently incorporated into DNA (DNA-6TGN). Cellular recognition of the resulting nucleobase mismatches induces apoptosis [3,8].

The improved ALL cure rates during the last decades suggest that many relapses in the past were insufficiently treated, partly due to variations in drug disposition, rather than reflecting treatment resistance.

### Individualized medicine and childhood acute lymphoblastic leukemia

for implementing individualized medicine the following issues need to be addressed:

#### 1. The patients exhibit variable treatment responses in terms of cure or side effects

If all patients responded uniformly to a disease treatment, an optimized standard treatment could readily be defined. This, however, is rarely the case. In ALL and other treatments involving drugs with low therapeutic indices, an important consequence of this variability is that standard doses must be set sufficiently low to avoid severe toxicities in the most sensitive patients including slow metabolizers, whereby some of the less sensitive remaining patients with more rapid drug elimination may be left under-treated. In cases where the inter-individual variation in drug disposition exceeds the intra-individual variation as well as the therapeutic window of the drug (more likely with drugs with low therapeutic indices), patients may benefit from individualized medicine [2].

#### 2. Diversity in cure/toxicity predictable by host genomics

In addition to outcome variability, patients must vary with respect to one or more geno/phenotypic marker that correlate with and therefore can be used to predict treatment responses. Generally, the outcome is determined by the sum of endogenous (primarily genetic) and exogenous effects (e.g. diet or prehydration prior to chemotherapy as well as unknown factors). The noise from the latter factors may significantly influence the intra-individual variation in drug disposition and thus hamper outcome prediction by genetic polymorphisms. When a major part of the genetic contribution to a specific outcome variability (e.g. a toxicity) can be ascribed to a single gene, a biphasic (or triphasic) frequency distribution of patients with respect to outcome may be observed. One such example is the role of TPMT status in myelotoxicity following 6MP therapy [5,10-12]. In contrast, when the response variation reflects the combined effect of multiple genes, a continuous frequency distribution of the outcome variability may be seen due to the presence or absence of many small contributions from the involved loci. In this scenario, patients may be at less well-determined risk of a specific toxicity and small variability in exogenous factors may significantly influence outcome. The response to high-dose MTX (HD-MTX) where toxicity reflects variability in both drug exposure (determined renal clearance [13] and hepatic metabolism [14] i.e. by variants in kidney and liver transporters and enzymes) and in drug sensitivity of target tissues (i.e. by apoptosis, DNA repair and MTX target gene variants [3,9]) is an examples of such multi-locus dependency.

Due to their simplicity, monogenic variants are easier understood in terms of underlying biology than multi-allele dependencies, but even for single-locus variants that seem strongly associated with a specific clinical effect, genetic linkage within haplotypes containing the true causal variant may cause misleading conclusions with respect to the biological mechanism of the gene-effect association. Causal biological understanding not only strongly increases the willingness for clinical application of a potential genetic marker for a biological outcome (item 7 below), but may also help in interpreting whether statistical associations reflect chance findings and point towards therapeutic interventions. With modern multi-locus genotyping techniques, the associations of thousands of variants with clinically defined variables can be tested, which easily leads to type I errors due to multiple testing. In hypothesis-based investigations, the higher probability of causality for the individual marker and especially the lower number of markers tested strongly diminish this risk, relative to random genome-wide association screenings. However, even a limited number of markers give rise to many testable genotype-outcome associations, when the markers are combined with each other and with multiple clinical outcome parameters and patient subgroups. Rocha *et al*. [15] investigated the effect of 16 genetic polymorphisms on hematological and CNS relapses in lower-risk (LR) and higher-risk (HR) patients in the St Jude protocol and reported several associations. However, many of these were only valid for selected combinations of risk group, anatomical relapse location and allele variants within in the remaining loci. Although correlation of two gene variants with the *in vitro *expression of their respective genes was clearly demonstrated and the authors provided plausible explanations why some genetic variants were mainly predictive in the HR group (more drugs and higher doses used), it is likely that at least some of the associations with the clinical endpoints are chance findings due to combinatorial multiple testing. Thus, in individualized medicine candidate associations limited to certain patient subgroup/marker/outcome combinations not only benefit fewer patients, but should also be confirmed more rigorously in independent experiments and populations.

#### 3. Treatment adjustments by genetic polymorphisms or therapeutic drug monitoring have predictable effects on efficacy/toxicity in individual patients

Predicting patients with unfavorable outcomes in response to standard treatment is of no use if adequate corrective measures cannot be made. For pharmacogenetic dose adjustments this implies that the desired or adverse effects correlate with the administered drug dose. Whereas individualized ALL therapy based on genetic markers this far has been limited to avoiding acute toxicities by adjusting 6MP doses by *TPMT *genotyping [12], attempts to improve ALL cure rates have relied on therapeutic drug monitoring. Evans *et al*. [16] reported improved cure rates of B-lineage ALL relative to standard doses, when MTX, teniposide and cytarabine doses were adjusted based on the patients' individual clearance rates of these drugs. This strategy was based on the following assumptions: I) Previously observed associations of relapse with lower drugs levels reflected causality (that is, lower drug levels is not a secondary bystander phenomenon following the true causal effect) and II) increasing drug doses would increase drug exposure and thereby reduce the relapse rate. Since multivariate analysis showed that exposure to MTX, but not to teniposide or cytarabine was associated with better chance of cure, the study suggests that both assumptions are valid for MTX. However, the individualized group did overall receive higher drug doses (and experienced more side effects) and it may therefore be argued that the outcome improvement is due to the overall treatment intensification rather than to the individualized approach per se. Nonetheless, the study clearly demonstrates that the individual approach was capable of identifying patients for whom the therapeutic potential of MTX was not fully utilized. Whether improved cure rates can be obtained while keeping the overall drug dosing constant (and thereby minimizing toxicity) still remains to be demonstrated. In a similar study in Nordic Society of Paediatric Haematology and Oncology (NOPHO) ALL-92 protocol, the patients' levels of 6MP and MTX metabolites (TGN·MTX product) in combination with bone marrow suppression (white blood cell and platelet counts) were used for 6MP and MTX dose adjustments during maintenance therapy of pre-B and T cell ALL [7]. Although the individualized group in this study did also receive higher drug doses (especially 6MP, which was primarily intensified to reach TGN·MTX target level), no outcome improvement was observed for the boys, and a 6.6-fold increase in relapse hazard was observed for the girls, relative to dosing by bone marrow suppression alone. The choice of 6MP as the primary drug to intensify was based on previous findings that TGN is more strongly associated with risk of relapse than MTX. However, increasing 6MP does not increase TGN, but rather elevates the levels of methylated 6MP metabolites [17,18]. Moreover, since high TPMT activity was associated with relapse it was speculated that higher levels of methylated 6MP metabolites put the leukemic cells in a dormant, chemoresistant state from which they expand after therapy discontinuation. Based on the finding in Evans' study [16] it might be speculated that better outcomes of the NOPHO patients would be achieved if MTX was the primary drug to intensify. Since 6MP dose increment did not increase TGN as predicted (assumption II) the effect of TGN levels on relapse rate (assumption I) became irrelevant in the context of this study. This does not mean that an individualized approach is not feasible. Provided that assumption I) is valid, adjustments such as co-treatment with TPMT inhibitors or adding 6-thioguanine to the 6MP therapy may correct low TGN levels and thereby improve the prognosis of these patients. This is currently being explored in the NOPHO cooperation.

#### 4. Dose adjustments by genetics better than by toxicity or by drug concentration measurements

During long-term continuous therapy or with repeated treatments, dose adjustment by concurrent measurements of drug concentration or clinical therapeutic targets (e.g. degree of myelosuppression during ALL maintenance therapy) may reduce the need for outcome prediction prior to treatment. For implementing dose adjustments by pharmacogenetics in such cases, it should be better than or add to dosing by drug monitoring or by toxicity. HD-MTX infusion over 24 hours with Leukovorin rescue is widely used in the treatment of childhood ALL [19], but patients vary substantially with respect to MTX elimination rates and steady-state concentrations [9]. Extremely delayed elimination with life-threatening MTX concentrations occur sporadically in repeated HD-MTX administration in individual patients and thus likely reflects exogenous factors (pre-hydration/alkalization etc.) rather than patient genetics [19,20], but more moderately delayed MTX elimination can to some extend be predicted by pharmacogenetics [21,24]. These patients receive extra Leukovorin doses in order to prevent toxicity, but this has been associated with an increased risk of relapse indicating rescue of leukemic cells [19]. Thus, since individualized HD-MTX dose adjustments by MTX measurements during-infusion may improve the cure rates [16], pharmacogenetically improved dosing of MTX could potentially reduce the inter-individual variations in MTX pharmacokinetics with a reduction in both undertreatment (too low MTX doses) an over-rescue (too high MTX concentrations). By analogy, *TPMT *genotyping is an example of how pharmacogenetic profiling can add to the individualization of therapy compared with toxicity-based guidelines, since for childhood ALL patients with TPMT low activity, the cure rate seems independent of the degree of myelosuppression obtained, whereas for TPMT high-activity patients 6MP/MTX dose adjustment during maintenance therapy to obtain myelosuppression seems to improve the cure rate [11]. Thus, pharmacogenetics is not an alternative, but a supplement to traditional dose adjustments by toxicity or drug measurements.

#### 5. Reducing toxicity or increasing efficacy must not be upset by less efficacy or more toxicity

The improved cure rates of childhood ALL over the last decades clearly demonstrate that many leukemias previously regarded treatment resistant were curable, if sufficiently intensive treatment is applied. Accordingly, dose adjustments to overcome adverse rapid drug elimination would be expected to increase cure rates further. However, life threatening or other unacceptable toxicities such as second malignant neoplasms after 6MP therapy [25] or avascular necrosis after glucocorticosteroid therapy [26] may preclude such intensification. Similarly, attempts to mitigate toxicities by dose reductions could increase the risk of relapse [27]. The impressive improvements in the cure of childhood ALL of today was obtained by general treatment intensification linked to improved risk grouping primarily based on characteristics of the leukemic clone (e.g. lineage, cytogenetics, and tumor burden). In recent years this has been refined by adding monitoring of the early response to induction chemotherapy (i.e. monitoring of minimal residual disease). However, due to the high frequency of toxicity, general treatment intensification seems unacceptable. Thus, although the additional 30% of the patients that is now cured compared to the 1970'ies certainly benefits from the increased intensity, the treatment is worse for the remaining 70%, since the 50% was already cured in the 1970'ies and the 20% that still fails has in general only experienced more side effects. This burden of toxicity is clearly reflected by the fact that 25-60% of deaths within 10 years after diagnosis of ALL are non-leukemic events [4,28]. Accordingly, pharmacogenetic identification of patients at risk of such toxicities and subsequent adjusting, including reducing, their treatment intensity may improve the overall survival of childhood ALL patients.

#### 6. Pharmacogenetic-kinetic data for individualized medicine should relate to relevant patient groups and treatment protocols

In theory, concordant findings should be obtained when significant associations are re-tested in independent patient populations, provided that the confirmatory study has appropriate statistical power. However, from the literature it is clear that supporting findings in association studies are less common [3,8,9]. Trivial explanations such as low sample sizes are often put forward, but additional leukemia and patient-associated factors are likely to be involved and should therefore also be integrated in genome-guided treatment adjustments [29]. As examples of the latter both Rocha [15] and Gregers *et al*. [21] have shown that the association between cure rates and pharmacogenetic variants may be restricted to specific ALL subsets. Thus, in a recent Danish study of 500 patients, the reduced folate carrier-1 (*RFC1*, involved in cellular MTX uptake) high activity-variant *RFC1 *A_80 _(rs1051266) [30] was associated with better event-free survival. However, the *RFC1 *gene is located on chromosome 21 and this genotype-phenotype association could not be shown in the subset of patients with three or more of chromosome 21 copies in their leukemic clone suggesting that a gene-dosage effect of the *RFC1 *may compensate for the lower activity of the G_80 _variant [21]. Since patients carrying the A_80 _variant also had higher plasma MTX levels, their superior outcomes may reflect an increased systemic exposure or higher sensitivity of target cell to MTX due to an enhanced cellular influx. In support of the latter interpretation, Buitenkamp *et al*. [31] reported that the higher treatment-related toxicity among ALL patients with Down syndrome after high-dose MTX most likely reflect higher sensitivity in affected tissues rather than higher systemic MTX exposure, since MTX clearance was only marginally (5%) lower among Down ALL patients and the severity of gastrointestinal toxicity was not related to systemic MTX exposure (AUC). Nonetheless, the slower MTX elimination was statistically highly significant, and since the *RFC1 *A_80 _variant may have a stronger impact on cellular MTX uptake and elimination rate than chromosome 21 trisomy, the findings by Buitenkamp [31] support the findings of by Gregers [21]. Altogether, these findings support that leukemia karyotype and congenital chromosomal syndromes [32] can both affect pharmaco-kinetics/dynamics and that this should be considered in the evaluation of response predictors to be used in treatment individualization.

Other examples of patient-associated confounding factors include ethnicity [33], age [34], and gender [35]. The latter is reflected in the inferior cure rates of girls in a therapeutic drug monitoring study of MTX/6MP maintenance therapy [7].

Finally, the effect of genetic variants on a drug is highly dependent on the treatment protocol. In its simplest form, divergent association findings may arise from differences in co-administered drugs that may diminish the role of the drug under investigation, or increase the expression of enzymes involved in catabolism of the drug in question (phenotype changes) [2,3]. Lavadiere *et al*. [22] reported that the *RFC1 *A_80 _variant was associated with higher plasma MTX concentrations during HD-MTX therapy in French Canadian ALL patients, which is in line with the findings by Gregers *et al*. in the NOPHO study [21]. In contrast, whereas *RFC1 *A_80 _was associated with lower event free survival in the Canadian study, significantly higher chance of staying in remission was observed in the Danish cohort. This discrepancy is likely to be explained by the more extensive use of HD-MTX in the Danish cohort (up to 9 courses of HD-MTX (5-8 g/m^2^/24h with i.t. MTX and 15 mg/m^2 ^Leucovorin rescue times three) during consolidation and maintenance therapy, whereas the Canadian patients only received one course of HD-MTX during induction therapy (4 g/m^2 ^MTX with 200 mg/m^2 ^initial Leukovorin rescue followed by 24 mg/m^2 ^per subsequent dose) [21,22]. The inferior (rather than similar) outcome among Canadian patients carrying the *RFC1 *A_80 _allele could potentially be ascribed to their higher plasma MTX concentrations and subsequent extensive Leukovorin rescue [19]. Whatever the cause, such conflicting findings emphasize the potential pitfalls when interpreting pharmacogenetic associations across patient groups and treatment protocols.

#### 7. The potential treatment adjustment is defendable statistically, biologically (well understood), and therapeutically

For genetic markers to be used in treatment individualization, their linkage to clinical outcome measures needs to be firmly established, and treatment adjustments to improve cure rates and reduce side effects should not only be statistically significant, but also validated in separate studies of independent patient populations that receive comparable therapy.

Ideally, the biology of genetic associations used in treatment individualization should be understood. Genome-wide association studies, how powerful they may be for identifying new genotype-phenotype associations, frequently lack clear biological explanations, but even for genetic variants that are clearly linked to well-described drug metabolism pathways, the mechanism of the association may be uncertain. Occasionally, drug concentration measurements may identify the underlying mechanism. In contrast, causality may be less obvious for associations with confounded (e.g. multi-drug treatments) or late clinical endpoints (e.g. event-free survival and late effects) or for markers that are only statistically linked (e.g. haplotype variants) with the causal variant. In principle, unexplained associations can justify treatment changes if the association is sufficiently strong. Thus, if host genomic profiling across study groups can identify subsets of patients highly resistant to conventional chemotherapy, shifting such patients to very intensive therapy, including bone marrow transplantation, may be as legitimate as risk grouping based on cytogenetic aberrations, such as for patients with Philadelphia chromosome positive ALL [36]. More likely however, genetic profiling will identify patients with moderately increased relapse risk, and the clinical decision-making (i.e. which treatment phase or drug to intensify) should be based on mapping of the association at the molecular level. An additional advantage of causal markers is that their clinical associations are more likely to be valid across ethnicity. Moreover, although an empiric approach may be scientifically/statistically justified, the lack of causal understanding may form a psychological barrier and hamper physicians' willingness to inflict further toxicity or risking reduced cure rates. Thus, treatment individualization is likely to be dominated by associations involving genetic variants that directly affect protein function or expression that can be further investigated at the functional level.

Identification of patients with high risk of poor outcome is of little use if alternative treatment is not available or acceptable. Fortunately for childhood ALL, higher drug doses and the associated toxicities seem acceptable for the antimetabolites, the glucocorticosteroids, and L-asparginase, but less so for vincristine, alkylating agents, topoisomerase-II inhibitors, and other DNA-damaging agents.

#### 8. The prospective risk profiling must be rapidly available and cost efficient

For implementing individualized medicine, there is a need to improve logistics that allow genotypic or phenotypic profiling (e.g. pharmacological measurements) within a reasonable time, which for drugs used during induction therapy may be as short as a few days. Owing to the low incidences of ALL and the specialized nature (not routinely available genetic analyses or pharmacological measurements) this likely involves centralized facilities. Since costs and logistics associated with such analyses, data registration and communication, and not least individual dosing to each patient by the clinicians is an elaborate task, the cost efficiency of such treatment individualization require careful evaluation. Table 1 shows the co-distribution of an adverse event and a risk marker in response to standard treatment in two hypothetical populations of 1000 patients each and with similar relative risks and odds ratios, but with different sensitivities (18% vs. 89%, respectively) and precisions (9.1% vs. 3.7%) in toxicity prediction. From a clinical point of view the costs of individualized medicine can be evaluated at three levels: I) Most simple by looking at the total patient population; does the frequency and severity of the adverse event (e.g. toxicity) justify the costs? This does not mean that toxicity prediction should be limited to frequent events, since very severe, although rare, toxicities may well justify the costs of genotyping. II) If so, how many of these toxic events among the total cohort can be avoided by treatment individualization. With the sensitivities in populations A and B, this is maximally 18 and 89%, respectively, provided that all toxic events are prevented by treatment individualization. The latter may not be the case and should therefore also be considered in the cost-benefit evaluation. III) Does the precision (9.1% and 3.7% of the high-risk patients, respectively, that benefits (potentially avoids toxicity) from intensity reduction) justify the loss (increased risk of relapse for the remaining 90.9% and 96.3% of the high-risk patients that did not experience toxicity with standard treatment) and how should these gains and losses be balanced? Certainly, the weighing should include both the number and the relative severity of the adverse events (e.g. ALL relapse vs. toxicity) expected with both standardized and individualized treatment, which will undoubtedly be a challenge. Unfortunately, most published papers on genotype-phenotype associations focus on relative risks or odds ratios rather than absolute risk and fraction of all events linked to a specific genotype, which is of more use in cost efficiency evaluations.

**Table 1:** The co-distribution of a risk marker (High/Low risk) and adverse event (+/- Toxicity) in response to standard treatment in two hypothetical patient populations (A, B).

Population A	Outcome	Low risk	High risk
RR/OR	- Toxicity	969	20
9.9/10.8	+Toxicity	9	2

Precision 2/(20+2): 9.1%		Sensitivity 2/(9+2): 18%	
			
**Population B**	**Outcome**	**Low risk**	**High risk**

RR/OR	- Toxicity	535	446
9.9/10.2	+Toxicity	2	17

Precision 17/(446+17): 3.7%		Sensitivity 17/(2+17): 89%	

Precision and sensitivity calculated as explained in the main text. RR: Relative Risk; OR: Odds Ratio

#### 9. Individualized medicine approaches should be tested in randomized trials

Regardless of statistical significance and degree of mechanistic understanding of genotype-phenotype associations, their clinical applicability should be tested in prospective randomized trials. Since the profile of toxicities in childhood ALL therapy is very wide and a limited number of patients available, it is unrealistic to perform randomized clinical trials for each toxicity, and furthermore difficult to obtain sufficient statistical power to demonstrate changes in the frequency of rare toxicities [37]. The division of patients into multiple risk groups and the late occurrence of many events further burden such trials. Still, addressing multiple toxicities and allowing genotype-based adjustments of several anticancer agents in order to reduce the burden of therapy and simultaneously improve cure rates may be a proof-of-principle approach even though the subsequent statistical and biological identification of the most important treatment modifications will be challenging.

## Conclusions

As initially stated, "individualized medicine" as a concept has gained popularity within the last decade - largely owing to the development of molecular techniques that allow patient genotyping in practically any laboratory. However, dose adjustments by therapeutic drug monitoring or bone marrow toxicity and stratification of patients to low- or high-risk treatment groups based on chromosomal aberrations, leukocyte counts at diagnosis, minimal residual disease, which has been performed for decades can also be considered "individualization", although traditionally not referred to as such. From this point of view, the improvement since the 1950´ies in childhood ALL therapy with overall survival rates rising from 50 to nearly 90% can be seen as proof that patients benefit from individualized medicine. However, the persisting high frequency of serious toxicities and relapses emphasize that implementation and further refinement of such strategies in leukemia treatment may be worthwhile.

## Competing interests

The author declares that they have no competing interests.

## Authors' contributions

All authors discussed and contributed to the content of the text. JN drafted the manuscript. All authors read and approved the final manuscript.

## References

[B1] Bates S Progress towards personalized medicine Drug Discov Today 2010 15 115 120 10.1016/j.drudis.2009.11.001 19914397

[B2] Gardiner SJ Begg EJ Pharmacogenetics, drug-metabolizing enzymes, and clinical practice Pharmacol Rev 2006 58 521 590 10.1124/pr.58.3.6 16968950

[B3] Davidsen ML Dalhoff K Schmiegelow K Pharmacogenetics influence treatment efficacy in childhood acute lymphoblastic leukemia J Pediatr Hematol Oncol 2008 30 831 849 10.1097/MPH.0b013e3181868570 18989161

[B4] Lund B Åsberg A Heyman M Risk Factors for Treatment Related Mortality in Childhood Acute Lymphoblastic Leukemia Pediatr Blood Cancer 2011 56 551 9 10.1002/pbc.22719 21298739

[B5] Schmiegelow K Bretton-Meyer U 6-mercaptopurine dosage and pharmacokinetics influence the degree of bone marrow toxicity following high-dose methotrexate in children with acute lymphoblastic leukemia Leukemia 2001 15 74 79 10.1038/sj.leu.2401986 11243403

[B6] Schmiegelow K Forestier E Hellebostad M Long-term results of NOPHO ALL-92 and ALL-2000 studies of childhood acute lymphoblastic leukemia Leukemia 2010 24 345 354 10.1038/leu.2009.251 20010622

[B7] Schmiegelow K Bjork O Glomstein A Intensification of mercaptopurine/methotrexate maintenance chemotherapy may increase the risk of relapse for some children with acute lymphoblastic leukemia J Clin Oncol 2003 21 1332 1339 10.1200/JCO.2003.04.039 12663723

[B8] Cunningham L Aplenc R Pharmacogenetics of acute lymphoblastic leukemia treatment response Expert Opin Pharmacother 2007 8 2519 2531 10.1517/14656566.8.15.2519 17931087

[B9] Schmiegelow K Advances in individual prediction of methotrexate toxicity: a review Br J Haematol 2009 146 489 503 10.1111/j.1365-2141.2009.07765.x 19538530

[B10] Relling MV Hancock ML Rivera GK Mercaptopurine therapy intolerance and heterozygosity at the thiopurine S-methyltransferase gene locus J Natl Cancer Inst 1999 91 2001 2008 10.1093/jnci/91.23.2001 10580024

[B11] Schmiegelow K Forestier E Kristinsson J Thiopurine methyltransferase activity is related to the risk of relapse of childhood acute lymphoblastic leukemia: results from the NOPHO ALL-92 study Leukemia 2009 23 557 564 10.1038/leu.2008.316 18987654 PMC3898327

[B12] Relling MV Gardner EE Sandborn WJ Clinical Pharmacogenetics Implementation Consortium (CPIC) guidelines for thiopurine methyltransferase (TPMT) genotype and thiopurine dosing Clinical Pharmacology & Therapeutics 2011 in press 21270794 10.1038/clpt.2010.320 PMC3098761

[B13] Skarby T Jonsson P Hjorth L High-dose methotrexate: on the relationship of methotrexate elimination time vs renal function and serum methotrexate levels in 1164 courses in 264 Swedish children with acute lymphoblastic leukaemia (ALL) Cancer Chemother Pharmacol 2003 51 311 320 12721759 10.1007/s00280-002-0552-1

[B14] Jacobs SA Stoller RG Chabner BA Johns DG 7-Hydroxymethotrexate as a urinary metabolite in human subjects and rhesus monkeys receiving high dose methotrexate J Clin Invest 1976 57 534 538 10.1172/JCI108308 1062383 PMC436681

[B15] Rocha JC Cheng C Liu W Pharmacogenetics of outcome in children with acute lymphoblastic leukemia Blood 2005 105 4752 4758 10.1182/blood-2004-11-4544 15713801 PMC1895006

[B16] Evans WE Relling MV Rodman JH Crom WR Boyett JM Pui CH Conventional compared with individualized chemotherapy for childhood acute lymphoblastic leukemia N Engl J Med 1998 338 499 505 10.1056/NEJM199802193380803 9468466

[B17] Erb N Harms DO Janka-Schaub G Pharmacokinetics and metabolism of thiopurines in children with acute lymphoblastic leukemia receiving 6-thioguanine versus 6-mercaptopurine Cancer Chemother Pharmacol 1998 42 266 272 10.1007/s002800050816 9744770

[B18] Lennard L Welch J Lilleyman JS Mercaptopurine in childhood leukaemia: the effects of dose escalation on thioguanine nucleotide metabolites Br J Clin Pharmacol 1996 42 525 527 10.1111/j.1365-2125.1996.tb00021.x 8904630 PMC2042700

[B19] Skarby TV Anderson H Heldrup J Kanerva JA Seidel H Schmiegelow K High leucovorin doses during high-dose methotrexate treatment may reduce the cure rate in childhood acute lymphoblastic leukemia Leukemia 2006 20 1955 1962 10.1038/sj.leu.2404404 16990760

[B20] Widemann BC Balis FM Kim A Glucarpidase, leucovorin, and thymidine for high-dose methotrexate-induced renal dysfunction: clinical and pharmacologic factors affecting outcome J Clin Oncol 2010 28 3979 3986 10.1200/JCO.2009.25.4540 20679598 PMC2940396

[B21] Gregers J Christensen IJ Dalhoff K The association of reduced folate carrier 80G > A polymorphism to outcome in childhood acute lymphoblastic leukemia interacts with chromosome 21 copy number Blood 2010 115 4671 4677 10.1182/blood-2010-01-256958 20335220 PMC2890175

[B22] Laverdiere C Chiasson S Costea I Moghrabi A Krajinovic M Polymorphism G80A in the reduced folate carrier gene and its relationship to methotrexate plasma levels and outcome of childhood acute lymphoblastic leukemia Blood 2002 100 3832 3834 10.1182/blood.V100.10.3832 12411325

[B23] Trevino LR Shimasaki N Yang W Germline genetic variation in an organic anion transporter polypeptide associated with methotrexate pharmacokinetics and clinical effects J Clin Oncol 2009 27 5972 5978 10.1200/JCO.2008.20.4156 19901119 PMC2793040

[B24] Hulot JS Villard E Maguy A A mutation in the drug transporter gene ABCC2 associated with impaired methotrexate elimination Pharmacogenet Genomics 2005 15 277 285 10.1097/01213011-200505000-00002 15864128

[B25] Schmiegelow K Al-Modhwahi I Andersen MK Methotrexate/6-mercaptopurine maintenance therapy influences the risk of a second malignant neoplasm after childhood acute lymphoblastic leukemia: results from the NOPHO ALL-92 study Blood 2009 113 6077 6084 10.1182/blood-2008-11-187880 19224761 PMC2699230

[B26] McNeer JL Nachman JB The optimal use of steroids in paediatric acute lymphoblastic leukaemia: no easy answers Br J Haematol 2010 149 638 652 10.1111/j.1365-2141.2010.08192.x 20408842

[B27] Pinkel D Hernandez K Borella L Drug dosage and remission duration in childhood lymphocytic leukemia Cancer 1971 27 247 256 10.1002/1097-0142(197102)27:2<247::AID-CNCR2820270202>3.0.CO;2-C 5100389

[B28] Saarinen-Pihkala UM Heilmann C Winiarski J Pathways through relapses and deaths of children with acute lymphoblastic leukemia: role of allogeneic stem-cell transplantation in Nordic data J Clin Oncol 2006 24 5750 5762 10.1200/JCO.2006.07.1225 17179109

[B29] Nersting J Schmiegelow K Pharmacogenomics of methotrexate: moving towards individualized therapy Pharmacogenomics 2009 10 1887 1889 10.2217/pgs.09.148 19958086

[B30] Baslund B Gregers J Nielsen CH Reduced folate carrier polymorphism determines methotrexate uptake by B cells and CD4+ T cells Rheumatology (Oxford) 2008 47 451 453 10.1093/rheumatology/ken073 18316334

[B31] Buitenkamp TD Mathot RA de H V Pieters R Zwaan CM Methotrexate-induced side effects are not due to differences in pharmacokinetics in children with Down syndrome and acute lymphoblastic leukemia Haematologica 2010 95 1106 1113 10.3324/haematol.2009.019778 20418240 PMC2895034

[B32] Taub JW Ge Y Down syndrome, drug metabolism and chromosome 21 Pediatr Blood Cancer 2005 44 33 39 10.1002/pbc.20092 15390307

[B33] Pui CH Sandlund JT Pei D Results of therapy for acute lymphoblastic leukemia in black and white children JAMA 2003 290 2001 2007 10.1001/jama.290.15.2001 14559953

[B34] Schmiegelow K Heyman M Gustafsson G The degree of myelosuppression during maintenance therapy of adolescents with B-lineage intermediate risk acute lymphoblastic leukemia predicts risk of relapse Leukemia 2010 24 715 720 10.1038/leu.2009.303 20130603

[B35] Pui CH Boyett JM Relling MV Sex differences in prognosis for children with acute lymphoblastic leukemia J Clin Oncol 1999 17 818 824 10071272 10.1200/JCO.1999.17.3.818

[B36] Arico M Valsecchi MG Camitta B Outcome of treatment in children with Philadelphia chromosome-positive acute lymphoblastic leukemia N Engl J Med 2000 342 998 1006 10.1056/NEJM200004063421402 10749961

[B37] Ansari M Krajinovic M Pharmacogenomics in cancer treatment defining genetic bases for inter-individual differences in responses to chemotherapy Curr Opin Pediatr 2007 19 15 22 10.1097/MOP.0b013e3280140613 17224657

